# Is Spinal Analgesia or Anesthesia Safe After Labor Epidural Analgesia? Reporting Two Cases of High Neuraxial Block and Mini-Review of the Literature

**DOI:** 10.3390/reports8030129

**Published:** 2025-08-01

**Authors:** Arsen Uvelin, Marijana Cavrić-Dragičević, Borislava Pujić, Lidija Jovanović, Teodora Tubić, Radmila Popović

**Affiliations:** 1 Clinic of Anesthesia, Intensive Therapy and Pain Treatment, University Clinical Center of Vojvodina, Hajduk Veljkova 1-10, 21000 Novi Sad, Serbia; marijana.cavric@kcv.rs (M.C.-D.); borislava.pujic@kcv.rs (B.P.); lidija.jovanovic@kcv.rs (L.J.); teodora.tubic@mf.uns.ac.rs (T.T.); radmila.popovic@mf.uns.ac.rs (R.P.); 2 Faculty of Medicine, University of Novi Sad, Hajduk Veljkova 3, 21000 Novi Sad, Serbia

**Keywords:** epidural analgesia, spinal anesthesia, Cesarean section, hypotension, respiratory insufficiency

## Abstract

**Background and Clinical significance:** Single-shot spinal anesthesia for intrapartum Cesarean section has recently been incriminated in carrying a high risk of high neuraxial block (HNB) occurrence in parturients receiving labor epidural analgesia. The so-called volume effect of the epidurally injected solution causes a contraction of the dural sack and unexpected HNB. **Case presentation:** We present two cases of HNB in parturients receiving epidural analgesia. The first case describes the 36-year-old patient G3P2, who was administered a repeated rescue analgesia single-shot spinal injection with low-dose local anesthetic (levobupivacaine, 3 mg) following non-functional combined spinal–epidural analgesia. The second case describes the 28-year-old parturient G1P0, who experienced HNB after single-shot spinal anesthesia with hyperbaric bupivacaine (7.5 mg) following labor epidural analgesia. **Conclusions:** Intrathecal administration of local anesthetic for the purpose of spinal analgesia or anesthesia in a parturient with epidural analgesia can cause unexpected HNB and could occur even at low doses of intrathecally administered medications. The interplay of numerous variables and circumstances in the specific case can result in the occurrence of HNB. We assume that in our first case, the volume effect and repeated dural puncture, and in the second case, the low height of the parturient coupled with the volume effect, played significant role in the occurrence of HNB.

## 1. Introduction and Clinical Significance

High neuraxial block (HNB) or high spinal anesthesia (HSA) is a recognized complication of neuraxial anesthesia/analgesia techniques that can be life-threatening. It is defined as a rapidly progressing motor block that is followed by respiratory paralysis, hypotension, and loss of consciousness [1]. Interestingly, the term total spinal anesthesia (TSA) is also used and refers to HNB causing respiratory failure and/or loss of consciousness, usually requiring airway intervention [2]. While some authors state that the term TSA is a misnomer and that the name HNB is the correct one, both are used in the literature [1,3]. Regardless of the name being used, HNB and its extreme presentation TSA are inherently associated with neuraxial anesthesia techniques and are among their most feared complications. It is important to stress that virtually every type of neuraxial technique (single-shot spinal anesthesia (SSSA), epidural anesthesia (EA), epidural analgesia (EAN), top-up EA after EAN, SSSA after failed top-up of epidural analgesia, SSSA for conversion to surgical anesthesia after EAN, combined spinal–epidural anesthesia/analgesia (CSE anesthesia/analgesia), and single-shot spinal analgesia (SSSAN)) carries the risk of HNB occurrence; the degree of the risk varies among the types of techniques, and some have been found to have an unacceptably high risk of HNB occurrence. The 7th National Audit Project of the Royal College of Anaesthetists of Great Britain identified 28 cases of cardiac arrest in obstetric patients receiving anesthetic care during a period of one year, of which 6 cases were attributed to HNB. One of those patients developed an HNB after the epidural top-up failure and a subsequent SSSA to provide rescue spinal surgical anesthesia for Cesarean section (CS) [4]. A Dutch study identified SSSA for intrapartum CS after EAN as a common cause of HNB [5]. On the other hand, the ESAIC guidelines state that SSSA is a valid anesthetic choice for intrapartum CS in a parturient already receiving EAN, with no clear recommendations on the doses of intrathecal medication [6]. Numerous case reports of HNB described in the literature are inconsistent in the case presentation, and many concomitant factors can play a distinct role in the appearance of HNB, especially when using SSSA for intrapartum CS in a parturient receiving EAN. We present two cases of HNB: the first one after a low-dose rescue SSSAN injection after the failure of CSE analgesia, and the second one after SSSA for intrapartum CS following EAN. The cases are unique in the sense that, until now, there have been no descriptions of HNB after such a low-dose local anesthetic given intrathecally for repeated SSAN and very-low-dose local anesthetic for intrapartum CS. Our two cases describe the interplay of variables that caused HNB and suggest further studies.

## 2. Case Presentation

### 2.1. Case 1

The 36-year-old G3P2, GW 39 + 5, weighing 95 kg and 163 cm tall, was admitted to the maternity ward of the Clinic of Gynecology and Obstetrics at the University Clinical Center of Vojvodina in Novi Sad, Serbia. The pregnancy was uneventful, the patient was healthy, and at the last check-up, two weeks before the admission, the fetal weight was estimated to be around 3300 g, with the head as the presenting fetal part. At about 4 cm cervical dilation, the parturient requested epidural analgesia, at the time having regular contractions without oxytocin stimulation. There was a plan to initiate CSE analgesia. In the sitting position, the spinal component of analgesia was administered, with the spinal puncture at the midline in the L3-4 interspace using a pencil-point 25 G needle (Pencan^®^, B. Braun, Melsungen, Germany) on the second attempt, and administering 2.5 mg of levobupivacaine (Levobupivacaine Kabi^®^ 5 mg/mL, FRESENIUS KABI NORGE AS, Halden, Norway) and 20 µg of fentanyl (Fentanyl^®^, Piramal Critical Care B.V., Voorschoten, the Netherlands) plus normal saline, to a total volume of 1.5 milliliters (mL). An epidural catheter was placed in the same position, through the same interspace, after a separate puncture and on the third attempt, using the loss of resistance to saline technique with a separate set (Perifix^®^, 401 Filter Set, B. Braun, Melsungen, Germany). The epidural space was located at a depth of 6 cm, and the catheter was threaded 5 cm into the epidural space without any problems and then secured and taped to the skin. When checking the epidural catheter, there was no free flow of any liquid when the outer tip of the catheter was placed below the puncture level, and there was no liquid of any kind aspirated from the catheter, with the two-milliliter syringe displaying negative pressure upon aspiration. The patient was positioned supine. At that moment she was still experiencing pain, described by her as a 6 on a numeric rating scale (NRS), with 0 being pain-free and 10 being the worst imaginable pain. She was describing her legs feeling warm, and she was able to lift the extended leg completely from the surface of the bed at the hip joint bilaterally. The patients are usually instructed to try to feel their skin with their fingers on the shoulder or the upper thorax, and then to try to feel the skin of both legs and the abdomen and thorax all the way up to the nipples, and describe if there are any changes in the sensation of light touch. At this time point, about 20 min had elapsed after the spinal analgesia administration, and the patient could not specify that any changes in the sensation of touch had occurred. Concerned that the pain was still high on the NRS, an epidural bolus of local anesthetic was administered through the catheter: 15 mL of 0.1% levobupivacaine with 2 µg of fentanyl per milliliter (administered as two aliquots of 7 and 8 mL, three minutes apart), and a basal epidural infusion was commenced at a rate of 10 mL/h with the same concentration (0.1% levobupivacaine with 2 µg/mL fentanyl). After 15 min (this was about 35 min after the start of CSE analgesia), the patient’s NRS pain score was 5. Hemodynamically, the patient was stable, her heart frequency was between 80 and 87/min, and her noninvasive blood pressure (NIBP)—measured every 5 min for the first 15 min period, then every 10 to 15 minutes in the next period—was 105–120/70–80 mmHg. After 35 min since the start of CSE analgesia, oxytocin stimulation was initiated. In about 10 min, the parturient complained of severe pain, with the NRS score being 8 or even 9. The attending anesthesiologist was concerned that the epidural catheter was not functioning at all, because the parturient felt no differences whatsoever in the touch sensation on the skin of her abdomen, perhaps only experiencing warmth in both of her legs, which were completely functional (Bromage 0). After 50 min since the commencement of CSE analgesia, with the epidural local anesthetic solution infusion already having been run for over half an hour, there was no improvement in pain scores. The anesthesiologist in charge suspected that the catheter was not positioned in the epidural space and chose not to administer another bolus of the LA/opioid mixture solution, but instead suggested that the patient withdraw the existing epidural catheter completely and re-site it, or administer a repeated spinal analgesia intrathecal injection at this point without even trying to place an epidural. The parturient opted for a repeated SSSAN injection. The patient was positioned in the left lateral decubitus and administered 3 milligrams of levobupivacaine and 10 µg of fentanyl in normal saline up to a volume of 1.3 milliliters intrathecally on the first attempt, through the L3-4 interspace and paramedian approach. Two minutes after the administration, the parturient complained that she was feeling dizzy. Immediately afterwards, she said that she felt like she was going to faint, and that she was feeling very weak. At the same time, she was not able to move her legs anymore, the Bromage scale being 3. Her HF dropped to 47/min, and the NIBP measurement was initiated but was unsuccessful, which was attributed to severe hypotension. Initially, 100 µg of phenylephrine and 0.5 mg of atropine were administered, followed by two more 100 µg phenylephrine boluses with about 20 s intervals and an epinephrine bolus of 10 µg. Another 300 µg of phenylephrine was added to Ringer’s solution that was being infused through a peripheral cannula. During this episode of hemodynamic instability, the fetal heart rate dropped to 90/min, the obstetrician checked for the signs of cord prolapse, and the parturient was administered oxygen via face mask (8 L/min). Only after about 90 s from the start of vasopressor administration, the NIBP measurement was successful and showed 67/43 mmHg. Gradually, in the next two minutes, the NIBP measurement showed the systolic pressure to be above 90 mmHg. The fetal heart rate recovered after about a minute and a half, to over 110/min. Subsequently, the cervical dilation continued, and in the next 120 min it reached 10 cm. The NRS score from the time point of the assumed HNB was 0 throughout the remainder of the labor. About 130 min after the HNB, the baby, weighing 3650 g, was born; the expulsion phase was uneventful. The total amount of phenylephrine was 900 µg, that of epinephrine was 20 µg, and that of atropine was 0.5 mg. Then, 150 min after the repeated spinal analgesia injection, the patient was able to move her legs, and she was transferred to the maternity ward along with her newborn. She was able to walk about three hours after the HNB and reported no headache on the following day, when she was discharged home along with the baby. Table 1 shows detailed information about Case 1 vital signs and clinical events.

### 2.2. Case 2

The 28-year-old G1P0, GW 40 + 6, weighing 55 kg and 152 cm tall, was transferred to a tertiary maternity clinic at the Clinic of Gynecology and Obstetrics of the University Clinical Center of Vojvodina, Novi Sad, Serbia, from a regional hospital in the afternoon hours. There was an attempt to induce general anesthesia for a category 3 Cesarean delivery, which was aborted, and the parturient was woken up, since the endotracheal intubation efforts were unsuccessful (glottic view Cormack–Lehane 4). Due to the lack of advanced airway management equipment, the attending anesthesiologist and obstetrician decided to transfer the patient to a tertiary unit. At arrival, after the initial obstetric exam, it was decided to try for a vaginal labor. After the start of induction with oxytocin, the dilation stage of the labor commenced. At about 4 cm dilation, the parturient was offered an epidural. An epidural catheter (Perifix^®^, 401 Filter Set, B. Braun, Melsungen, Germany) was placed through the L3-4 interspinous space after the first attempt, via the median approach, with the catheter being 5 cm in the epidural space. A test dose of 10 mL of 0.1% levobupivacaine with 2 µg/mL fentanyl (5 mL + 5 mL increments, three minutes apart) was administered. Immediately after the epidural bolus, the epidural 0.1% levobupivacaine with 2 µg/L fentanyl infused at 8 mL/h was started. The NRS after 20 min was 4, and the sensation to light touch reached dermatomal level Th 9. The oxytocin drip was recommenced, after which the pain increased from 4 to 7, with the contractions being felt predominantly on the right side, beneath the umbilicus and in the right groin. The catheter was withdrawn 1.5 cm from the epidural space, aspirated with no liquid upon aspiration, and 5 mL of 0.25% levobupivacaine solution was injected epidurally. Then, 20 min after the local anesthetic bolus, the pain score was 2, which was graded as satisfactory pain control. The continuous epidural solution was recommenced at a rate 10 mL/h. After another four hours, the cervical dilation stopped at 8 cm, and there were decelerations on cardiotocography. With the fetus weight estimated at around 3600 g, without the head engaging the pelvis, it was decided that the vaginal labor should be stopped and the baby delivered via a CS (category 2 of urgency). At that time point, the epidural continuous infusion had been administered for about four and a half hours, and the contractions were frequent, with another labor pain increase on the NRS, from 2 or 3 to 8. The anesthesiologist decided to top up the epidural catheter and convert EAN to surgical anesthesia. It was planned for the parturient to be administered 10 mL of 2% lidocaine with 2.5 µg/mL epinephrine and another 6 mL of 0.5% levobupivacaine in three aliquots. During the initial lidocaine epidural bolus, the patient declared that she felt the liquid going down her back but only feeling it like it was being infused in the left side of her lower back, where the epidural puncture site was. At that moment, with 4 mL of 2% lidocaine administered through the epidural catheter, the epidural top-up was aborted, and a decision to opt for an SSSA was made. Since the patient experienced the breakthrough pain on two occasions during the labor, it was assumed that the epidural analgesia was only partially functioning, and that the conversion would probably not result in satisfactory surgical anesthesia. In the operating room, the epidural catheter was withdrawn completely (the depth of the catheter was 7.5 cm at the skin level, which would have meant that it was still sitting in the epidural space), and spinal anesthesia was administered in the sitting position. A 7.5 mg hyperbaric solution of bupivacaine (Marcaine^®^ Spinal 0.5% Heavy 5 mg/mL, Cenexi, Fontenay-sous-Bois, France), 15 µg of fentanyl, and 100 µg of morphine (Morfin, Alkaloid, Skopje, Northern Macedonia) were injected intrathecally on the first attempt, using the median approach, in the L3-4 interspace, with a 25 G pencil-point spinal needle (Pencan^®^, B. Braun, Melsungen, Germany). The hemodynamic variables from initiation of the case until the parturient left the operating room are shown in Table 1. The baby was delivered exactly 10 min after the SSSA administration, with Apgar scores of 7/9. After 15 min into the procedure, with the estimated blood loss being around 400 mL, the parturient experienced having trouble breathing and swallowing. The anesthesiologist tested her touch, and the patient declared that she was feeling numb, below the dermatome level of Th2. At that time point there was a high suspicion of HNB development. The hemodynamic variables were as follows: NIBP 85/60 mmHg, HR drop from 97 to 81/min. At this moment, 100 µg of phenylephrine, 10 µg of epinephrine, and two 0.4 mg bolus doses of atropine were administered. In the next 2 min, the heart rate was 80/min, 0.4 mg of atropine and another 100 µg bolus of phenylephrine were given, and 400 µg of phenylephrine was added to Ringer’s solution that was being infused through a peripheral cannula. Diagnosis of local anesthetic systemic toxicity (LAST) was also considered and, although highly unlikely, it was decided to infuse a 20% fat emulsion as lipid resuscitation (Intralipid™ 20% 100 mL, Fresenius Kabi AB, Uppsala, Sweden) during the next 15 min. The CS was completed after 22 min of SSSA injection. The parturient remained in the operating room for 75 min after the spinal block. At discharge, her vitals were stable: NIBP 110/64, HR 103/min, without vasopressor infusion, and SpO_2_ 99% breathing room air and she felt light touch at Th 6 skin dermatome. She was able to move her legs three hours after the SSSA. Table 2 presents the timeline of clinical events and therapeutic measures in the second case. The total amount of phenylephrine infused was 750 µg, with 10 µg of epinephrine and 0.8 mg of atropine.

## 3. Discussion

Both cases describe the intrathecal administration of a local anesthetic/opioid mixture in a parturient already receiving a neuraxial technique for pain alleviation during labor. The first case describes a repeated SSSAN injection after a poorly functioning CSE analgesia technique. The parturient was experiencing severe labor pain after the administration of CSE analgesia, with the NRS never below 5. What was troubling to us while managing this case was that the spinal component of the CSE analgesia appeared to be providing unsatisfactory alleviation of the labor pain. The appearance of breakthrough pain could signify a poorly functioning epidural or a dysfunctional labor [6,7]. It seems that the local anesthetic requirements are higher in cases with dystocia [7]. A non-functioning or failing epidural was defined by Thangamutu and colleagues as an epidural with one or more of the following characteristics: lack of adequate pain relief 45 min after the initiation of EAN, dural puncture, re-siting or abandoning the epidural, and maternal dissatisfaction with analgesia [8]. Non-functional analgesia could have resulted from a faulty administration of the LA/opioid mixture, for example, if the spinal needle was displaced after the syringe was attached to it and the parturient never received the total dose (or any of the drugs). This was unlikely, since the operator had experience in the technique. The non-functional epidural could have resulted from the usage of the technique for CSE analgesia that uses two punctures, i.e., the spinal analgesia was administered through the spinal needle, and after that the epidural space was located separately and the catheter was threaded through a standard epidural needle. The CSE technique that uses a needle-through-needle technique offers a higher success rate for the location of the true loss of resistance from the needle being in the epidural space, since puncture of the dura with the spinal needle and the free flow of the cerebrospinal fluid confirm that the intrathecal space is in the very near vicinity [9]. The deviation from the original needle-through needle technique in our case was one more reason for the anesthesiologist to suspect that the epidural catheter was not functioning and that there was a false loss of resistance that occurred during the location of the epidural space, along with the fact that it was detected on the third attempt. The technique described here was used because there were shortages in the supply of the original sets for needle-through-needle techniques. Due to the difficulty encountered in locating the epidural space and the fact that the patient was a multipara, the second SSSAN was offered as rescue analgesia, since the parturient refused epidural catheter re-siting. Sharpe and coworkers described a retrospective cohort of 428 parturients who were administered SSSAN for the purpose of labor analgesia. Only 14% of these patients required additional labor analgesia, and some of them were given CSE or a second SSSAN injection [10]. SSAN can be regarded as the intrapartum analgesia of choice in low- and middle-income countries. Keser and coworkers used SSAN as the sole technique for labor analgesia, and after approximately 110 min of spinal analgesia duration, two-thirds of the parturients delivered, and most of them were satisfied with their labor analgesia [11]. What struck us while managing our case was the occurrence of profound hypotension and hemodynamic compromise after such a small dose of the LA/opioid mixture that is normally used for spinal labor analgesia. We believe that this was the case of HNB, and that the presence of anesthesiologist by the side of the patient and swift therapeutic measures prevented a worse clinical scenario. We postulate that multiple attempts to locate the epidural space and even unrecognized dural puncture, together with the volume effect of the epidurally infused LA/opioid mixture, caused the cephalad movement of the second administered intrathecal dose. The diffusion of the epidurally aggregated LA/opioid solution into the subarachnoid space through at least two dural punctures could have had a significant additive effect in the occurrence of the HNB. The volume effect was described back in 1997 by Japanese authors and entails a contraction of the dural sac caused by the local anesthetic solution, which is infused into the epidural space and compresses the dural sac from the outside [12]. This causes the intrathecally administered drugs to move more cephalad than expected, thus causing a higher spinal cord blockade. Epidural volume extension (EVE) as a technique that modifies CSE anesthesia has arisen from the volume effect of epidurally administered solutions. During EVE, a solution (which can be normal saline) is injected into the epidural space after the intrathecal block to increase the cephalad movement of the intrathecally administered medication [13]. EVE reduced the incidence of visceral pain during CS and improved patients’ satisfaction [13]. In another study, patients undergoing proximal femoral surgery who were administered EVE had a significantly quicker onset of sensory and motor block and prolonged duration of sensory block [14]. The hallmark of our case was the hypotension and bradycardia, along with the sudden motor block of the lower extremities, developing in less than two minutes after the repeated SSSAN injection. For the first three minutes after the spinal analgesia intrathecal injection, NIBP measurements were unsuccessful, in our opinion, due to profound hypotension; the parturient was drowsy and had a palpable faint brachial pulse that was bradycardic. After 300 µg of phenylephrine, the first measured NIBP was 67/43 mmHg. The fetal bradycardia was attributed to profound maternal hypotension and recovered quickly after the blood pressure stabilized. Our patient did not experience respiratory insufficiency, although the definitions of HNB and TSA include respiratory arrest as a hallmark. We believe that this was the cause of HNB, with severe hemodynamic compromise as the main sign, although one could advocate that, without respiratory insufficiency, the differential diagnosis might include transient vasoplegia after sympathetic block and vasovagal reflex. Radwan and coworkers, in their narrative review, addressed the clinical picture of TSA by describing the 51 cases of TSA retrieved from the literature. They stated that hypotension was present in nearly half of the cases, while 78% of the HNB parturients had to be endotracheally intubated [2]. Kuczkowski described a case of respiratory arrest in a parturient with a poorly functioning epidural catheter for analgesia, which was replaced with CSE analgesia [15]. After 4 min of spinal analgesia injection with low-dose bupivacaine and fentanyl, the parturient experienced respiratory arrest. The author postulated that the cephalad movement of the fentanyl due to contraction of the epidural space caused respiratory depression. HNB was not mentioned as a differential diagnosis, although there was hypotension present; the details of that case are summarized in Table 3. The signs and symptoms of the HNB development were swiftly addressed with appropriate therapy, which, in our opinion, prevented the progression to cardiocirculatory arrest. Immediate availability of an anesthesiologist who is able to address any complications of neuraxial analgesia techniques being administered is paramount.

In the second case, it surprised us that such a small dose as 7.5 mg of bupivacaine caused HNB. The characteristic of the HNB was that the clinical picture developed gradually, and the diagnosis of HNB was made 15 min after the spinal medication injection. SSSA in this case was a justified anesthesia technique for the provision of surgical anesthesia for intrapartum CS. The ESAIC’s focused guidelines issued a recommendation that “each instance of failed augmentation of labor epidural analgesia for intrapartum cesarean delivery should be addressed on an individual basis and that any of the both neuraxial anesthesia (including epidural top-up, new spinal or combined spinal-epidural techniques) and general anesthesia may be appropriate choices” [6]. At the end, it is stated that this is a conditional recommendation, and that the quality of the evidence is very low. It was the decision of the attending anesthesiologist to perform SSSA after the epidural catheter was evaluated as being poorly functional and the judgment that there was not enough time for a re-siting. It must be remembered at this point that the patient had a history of difficult endotracheal intubation, and that there was only one anesthesiologist available at 11 pm. The scenario of a non-functional epidural anesthesia during CS was not acceptable. A national survey of British anesthesiologists addressed the issue of the decision-making process in the scenario of converting epidural analgesia to anesthesia in an online national survey [16]. This study is important in that it shows that very high numbers of anesthesiologist would decide to perform SSSA as the anesthesia of choice after a failed top-up of the existing epidural catheter. In this survey, anesthesiologists, whether trainees or consultants, were asked what anesthetic technique they would use after the failure of a top-up of the existing epidural catheter for labor pain, depending on what clinical picture they encountered (namely, no objective sensory block, bilateral T10 dermatomal level, or unilateral T6 dermatomal level of sensory block). For example, if, after an epidural top-up, there was no objective sensory block, 74% of the anesthesiologists would perform SSSA as a rescue technique. On the other hand, Furst and colleagues described a small cohort of parturients who were administered SSSA after a failure of epidural anesthesia, and they reported 11% incidence of HNB, compared to 0.2% incidence in patients receiving only spinal anesthesia, and concluded that if there is a failure of top-up epidural anesthesia, SSSA should not be used as a rescue anesthesia technique for the provision of surgical anesthesia [17]. Carvalho specifically opposes the single-shot spinal technique for CS in the setting of failed top-up of the existing epidural catheter, because, as he emphasizes, it can be very difficult or even impossible to predict the correct dose of local anesthetic for intrathecal administration, and a much better option would be CSE anesthesia [18]. What about patients receiving epidural analgesia in whom there is the need for intrapartum CS, and specifically, in whom there have been no attempts at epidural top-up? Is SSSA a choice for these parturients? There is an abundance of case reports described in the literature, but the only consistent information that is reported is the dose of the local anesthetic intrathecally and the interspace through which the SSSA was delivered. Table 3 quotes several case reports from the literature addressing the occurrence of HNB in parturients who had epidurals running when the indication for intrapartum CS was given [15,17,19,20].

**Table 3:** Selected examples of case reports in parturients receiving epidural analgesia and subsequent single-shot spinal anesthesia for intrapartum Cesarean section with similar presentations of high neuraxial block.

Author, Year	Description of the Incident	Dosage of the Local Anesthetic/Opioid Mixture	Outcome and Possible Explanation
Kuczkowski KM, 2002 [15].	Poorly functioning EAN for labor pain requiring two rescue boluses, epidural catheter removed and replaced with CSE analgesia (spinal injection with low-dose fentanyl, low-dose bupivacaine) 4 min after spinal analgesia injection desaturation, loss of consciousness, NIBP 75/40, fetal heart rate drop to 60/min, intubation and supportive treatment. After 30 min, extubated. Vaginal delivery in progress, the epidural continued with good pain relief. Due to the failure to progress, proceeded to intrapartum CS in epidural top-up anesthesia, without complications.	Bupivacaine **2.5 mg** + fentanyl 10 µg	Respiratory insufficiency attributed to cephalad fentanyl movement. No mention of high neuraxial block, although there was a significant drop in blood pressure. No change in motor function of the lower extremities; good neonatal outcome.
Gupta A. et al, 1994 [19].	Poorly functioning EAN; SSSA for a CS due to failure to progress; five minutes after the intrathecal injection, difficulty in breathing, apnea, hypotension; endotracheal intubation, and conversion to general anesthesia.	Hyperbaric bupivacaine 0.5% (8% dextrose) **12.5 mg**	Extubated at the end of the CS, good neonatal outcome.
Gupta A. et al, 1994 [19].	Well-functioning EAN; SSSA for CS due to failure to progress; two minutes after intrathecal administration of LA, difficulty in breathing and apnea, “slight fall of blood pressure”, endotracheal intubation, and conversion to general anesthesia.	Hyperbaric bupivacaine 0.5% (8% dextrose) **10 mg**	Extubated at the end of the CS, good neonatal outcome.
Virgin H. et al, 2016 [20].	Poorly functioning EAN–intermittent bolus. Due to failure to progress, indication for intrapartum CS. SSSA as anesthesia of choice. One minute after the intrathecal injection, signs of motor weakness in the upper extremities, respiratory insufficiency, desaturation and loss of consciousness, and conversion to general anesthesia. About 20 min after the SSSA, the patient was breathing spontaneously but kept intubated and underwent a CT scan of the brain and the thorax to rule out pulmonary embolus or cerebral insult. After this was ruled out, she was extubated.	Hyperbaric bupivacaine 0.5% (8% dextrose) **13 mg** + fentanyl 25 µg + morphine 100 µg	No detailed information about the hemodynamics, described as a possible total spinal anesthesia; the last bolus of epidural solution 135 min before the performance of the SSSA; good neonatal outcome.
Furst SR and Reisner LS, 1995 [17].	SSSA for intrapartum CS after failure of full-dose top-up of existing epidural catheter; 5 min after the spinal block, respiratory insufficiency, difficulty in breathing, motor weakness in upper extremities, endotracheal intubation, and conversion to general anesthesia; 50 min after the spinal block, the Cesarean delivery was complete, and the patient began breathing spontaneously and was extubated.	Hyperbaric bupivacaine 0.75% **12 mg** with morphine 0.3 mg; spinal block at L2-3	No information about the hemodynamics; the case was described as a high spinal block; good neonatal outcome.
Furst SR and Reisner LS, 1995 [17].	Small cohort of parturients (27 patients) with failed epidural anesthesia who underwent subsequent SSSA administration as a rescue technique for achieving surgical anesthesia; 3 out of 27 patients experienced HNB. No detailed information was given as to whether these patients underwent intrapartum Cesarean section and already had an in situ epidural catheter. This cohort of patients was compared to the group of 643 patients who were administered spinal anesthesia as a single technique. One patient in this group experienced HNB.	Mean spinal bupivacaine dose in the three patients who developed high spinal block was **12 mg** vs. 12.2 mg in the remaining patients without high spinal block	

When comparing the cases from the table, the dose of intrathecal hyperbaric bupivacaine was above 10 mg in all instances, and the characteristics of the descriptions show that much information was lacking; for example, in some case reports, the information about the parturients’ height and weight was missing, and this should have never been omitted. A Dutch nationwide study from 2024 reported five cases of HNB during a 2.5-year period of nationwide surveillance, three of which occurred after SSSA for intrapartum CS in parturients with EAN for labor [5]. The investigators defined HNB as a need for ventilator support or cardiopulmonary resuscitation immediately after the performance of a neuraxial technique. The details of the three cases state the dosages of local anesthetic and the height of the spinal puncture. In one case, the spinal anesthesia procedure was performed at the L1-L2 level, which is unacceptable, and with a 10 mg dose of hyperbaric bupivacaine. The second case states L3-L4 puncture with 12.5 mg of hyperbaric bupivacaine, while the third does not cite the level of spinal puncture but states the dose of local anesthetic (articaine), which was 60 mg. The doses used in all of the cited case reports were higher than the dose used in our case, which was 7.5 mg. A Korean study researched this topic in a prospective randomized trial, using 10 mg of hyperbaric bupivacaine with 15 µg of intrathecal fentanyl in parturients randomized to receive SSSA for intrapartum CS following epidural labor analgesia [21]. The control group received epidural top-up anesthesia. They found no statistically significant differences in the frequency of HNB, i.e., only one patient receiving SSSA after EAN experienced HNB.

Visser and coworkers presented a retrospective cohort of parturients receiving SSSA following labor EAN. No differences with respect to the occurrence of HNB were found between the groups of parturients receiving SSSA for intrapartum CS following labor EAN compared to those receiving SSSA only [3]. Interestingly, the authors mentioned both high and total spinal anesthesia, with HNB defined as a patient complaining of impaired breathing but not requiring intubation, as opposed TSA, which, apart from respiratory insufficiency, included loss of consciousness requiring endotracheal intubation. Since it was a retrospective study, the data retrieved showed that anesthesiologists used a wide range of LA—namely, from 1.5 mL to 3 mL of hyperbaric or plain bupivacaine solution with or without sufentanil—and at the end concluded that there were no differences in the incidence of serious side effects, namely, high and total spinal block.

Surprisingly, we were able to find only one study that addresses the institutional guidelines on the application of SSSA as the anesthesia of choice for intrapartum CS in a parturient with a poorly functioning epidural catheter. Dadarkar et al. used SSSA in this patient group in a specific manner and with strict contraindications [22]. When SSSA was considered, epidural continuous infusion was continued up to the patients’ arrival in the operating room, but epidural boluses were avoided for at least 30 min prior to the SSSA procedure, the highest spinal bupivacaine dose was reduced from the usual maximum 12 mg to 10.5 mg, and supine positioning following intrathecal injection was delayed. SSSA was not considered if the catheter had been bolused in the preceding 30 min, if the parturient weighed more than 120 kg, if her height was less than 147 cm, or if there was an accidental dural puncture during the epidural catheter placement. In their cohort of 115 parturients, the median bupivacaine dosage was 9.38 mg, and there were no cases of HNB. If we apply these institutional guidelines to our two cases, then the first case is troublesome on the grounds of the volume effect, in that the parturient underwent repeated intrathecal injection. No matter how small the second intrathecal dose was (3 mg levobupivacaine + 10 µg fentanyl), it was enough to cause the HNB. Some would dispute that this was the cause of HNB, since the respiratory signs were scarce; nevertheless, it was certainly the cause of a profound hemodynamic instability that was life-threatening. If Dadarkar’s guidelines for SSSA were applied to the second case, then the bolus of 4 mL of 2% lidocaine with epinephrine, which could have been described as an epidural test dose, actually contraindicated the SSSA in our patient. As for the lipid resuscitation that was administered in case of the highly unlikely event of LAST in our case, the anesthesiologist postulated that it could only be of benefit to the patient, with no deleterious effects. There are very strange presentations of LAST described in the literature, and for that reason, lipid administration was administered [23]. Did the lipid infusion contribute to the redistribution of the LA from the nerve tissue and help resolve the HNB? We cannot know for sure, but this is probably unlikely.

We postulate that the extreme height, which was on the lower end of the mentioned guidelines, and the preceding epidural bolus were probably the reasons for HNB occurrence in our parturient.

Finally, we must express some doubts about the quality of the cases’ management. In the first case, the shortage of the original CSE kits did not warrant the use of a modified technique with two punctures. Multiple punctures or passes through tissue carry a higher risk of inadvertent or unnoticed dural puncture and can cause higher infection rates. At the same time, this modified technique was the cause of the suspicion that there was a false loss of resistance that occurred and that the catheter was not sitting in the epidural space at all. The repeated SSAN injection after a difficult epidural location and multiple previous tissue passes is also questionable. Would a simple epidural have sufficed, or would abandoning the technique and opting for a remifentanil infusion have been a better solution for the parturient? The second case could also have been handled differently. As a category 2 Cesarean section, the anesthesiologist still had enough time for epidural re-siting or the selection of CSE anesthesia instead of SSSA.

At the same, it is important to recognize that intrapartum CS in a parturient warrants special consideration concerning the choice of anesthesia. Slovenian authors described a clear relationship between labor analgesia modalities and types of anesthetic techniques in category 2 and 3 intrapartum Cesarean deliveries. In their small cohort of patients, most of the parturients with EAN were successfully converted to EA, and SSSA was the type of anesthesia most commonly used in the group of parturients who opted for patient-controlled analgesia with remifentanil [24]. Does this mean that the choice of labor analgesia suggests the anesthesia for intrapartum CS? The ESAIC guidelines still leave a wide choice for the type of anesthetic in intrapartum CS in cases where the augmentation of the labor EAN has failed, and they state that there is very low-quality evidence on the risk of HNB when a new SSSA is performed after epidural labor analgesia [6]. In parturients with an epidural catheter, the anesthesiologist should actively seek epidurals that are poorly functional and re-site and test them early on in the course of the labor, so that the catheter can be used for converting epidural analgesia to surgical anesthesia if necessary.

## 4. Conclusions

Ultimately, we conclude that intrathecal administration of local anesthetic for the purpose of spinal analgesia or anesthesia in a parturient with EAN can cause an unexpectedly high neuraxial block and could even occur at such low doses as mentioned in our two cases. The interplay of numerous variables and circumstances in the specific case can result in the occurrence of HNB.

The clarification as to whether to perform spinal anesthesia in a parturient with an existing epidural catheter is of absolute necessity, because the current guidelines are very general and the recommendations are vague. In our opinion the questions to be answered about intrapartum CS anesthesia choice are as follows:

1. Is SSSA a valid choice in a parturient already receiving an EAN for labor pain if the need for CS has occurred?
2. What are the doses of intrathecal LA in SSSA after EAN? Are they much lower than assumed? How low can we go without risking the occurrence of a failed SSSA?
3. Are there any contraindications—for example, a wet tap during the initial epidural catheter placement, a dural puncture due to an already performed CSE analgesia technique, multiple attempts during the initial epidural catheter placement, extremes in the height of the patient (i.e., short parturients), or boluses of epidural solution as rescue pain relief immediately before the SSSA?
4. If there is a failure of epidural top-up, should SSSA be considered as a rescue anesthesia technique at all? We postulate that too many variables exist and, in these instances, the dose of LA is unpredictable, and CSE anesthesia should be the preferred technique.

We call for a multicentric study that would give answers to all of these questions. The obstetric anesthetic practice is not the same throughout the Europe. There are centers that probably have much more experience in repeated, double-sequential neuraxial techniques and should be recruited for the purpose of further study. The guidelines should be very clear on this issue and be unequivocal.

## Figures and Tables

**Table 1:** Timeline of the first case, with hemodynamic and respiratory status variables and treatment.

Time After Intrathecal Administration of LA/Opioid Mixture, CSE Analgesia Start	NIBP [mmHg]	HR Beats/min	SpO_2_ [%] on Room Air	NRS (Numeric Rating Scale; 0—No Pain, 10—The Worst Imaginable Pain)	Bromage Scale (0–3)	Changes in Sensation of the Skin to Light Touch	Clinical Events	Vasopressor Therapy
The first vitals measured, immediately before spinal block	110/67	72	98%	10		Absent	Spinal component of CSE: 2.5 mg levobupivacaine + 20 µg fentanyl	
20 min	115/74	80	98%	6	0	Absent	15 mL 0.1% levobupivacaine epidural bolus in two aliquots; start of continuous epidural infusion of 0.1% levobupivacaine with 2 µg/mL fentanyl (10 mL/h)	
35 min	114/72	82	99%	5	0	Absent, or just feeling the warmth in the legs	Start of oxytocin infusion	
45 min	130/84	88	99%	8–9	0	Absent		
50 min				9	0	Absent		
55 min			99%,		0	Absent	Repeated single-shot spinal analgesia injection: 3 mg levobupivacaine + 10 µg fentanyl	
57	Not successful	47 on ECG	Lost trace	Not evaluated	3	Not evaluated	Fetal bradycardia 80–90/min	Phenylephrine 100 µg + 100 µg + 100 µg + atropine 0.5 mg + epinephrine 10 µg; 300 µg phenylephrine added to Ringer’s solution
60	91/56	95	99%, face mask with O_2_ 8 L/min	0	3	Numbness to the lower edge of the sternum	Fetal heart rate > 110/min	
75	103/72	87	99%	0	3	Numbness to the lower edge of the sternum		
175	105–125/73–81	75–87	99%	0	3	Numbness at umbilicus	Cervical dilation 10 cm	Vasopressors were administered 60 min after the HNB; hte total phenylephrine dose was 900 µg
185	110/70	84	99%	0	3		Delivery	
210–220	115/72	80	98%	-	2		Transfer to maternity ward	

Abbreviations: NIBP—noninvasive blood pressure, HR—heart rate, SpO_2_—saturation of peripheral blood with oxygen, NRS—numeric rating scale for pain objectivization: 0—no pain, 10—the worst imaginable or experienced pain; Bromage scale of assessment of motor block in the lower extremities: 0—no block, patient can lift the leg from the surface; 1—cannot lift the leg, but can flex the leg at the knee; 2—cannot flex the knee, but can move the feet; 3—complete motor paralysis of the legs.

**Table 2:** Timeline of the second case, with hemodynamic and respiratory status variables and treatment.

Time Point After the Intrathecal Administration of LA/Opioid Mixture	NIBP (Systolic/Diastolic) [mmHg]	HR Beats/min	SpO_2_ [%]	Clinical Events	Vasopressor Therapy
The first vitals measured, immediately before spinal block	140/75	105	99% room air		
4 min	90/50	95	98% room air		Phenylephrine bolus 50 µg, blood pressure drop due to spinal block
6 min	100/53	97			
15 min	85/60	81	96% room air	Parturient having trouble breathing and swallowing, light touch felt on the skin at Th2 dermatome	Bolus phenylephrine 100 µg, epinephrine 10 µg, atropine 0.4 mg + 0.4 mg (three minutes apart), phenylephrine 400 µg added to Ringer’s solution Start of Intralipid infusion Remark: epinephrine accelerates HR from 81 to 90/min, followed by another drop to 83/min; atropine 0.4 mg accelerates HR from 83 to 89/min and from 84 to 97/min
22 min	97/47	89	100% supplemental oxygen 6 L/min via face mask	Cesarean section completed >Apgar	Bolus phenylephrine 50 µg + 50 µg apart from phenylephrine infusion running
23–51 min	95–100/45–55		99–100%, O_2_ 6 L/min	Having trouble swallowing	
52	87/43	84	98% room air	Phenylephrine continuous infusion completed, another drop in NIBP	Another 150 µg of phenylephrine added to Ringer’s solution
60	105/53	98	99% room air	Light touch on the skin felt at Th 6 dermatome	
75	110/64	103	99% room air	Discharge to obstetric high-dependency unit	

Abbreviations: NIBP—noninvasive blood pressure, HR—heart rate, SpO_2_—saturation of peripheral blood with oxygen, NRS—numeric rating scale for pain objectivization: 0—no pain, 10—the worst imaginable or experienced pain; Bromage scale of assessment of motor block in the lower extremities: 0—no block, patient can lift the leg from the surface; 1—cannot lift the leg, but can flex the leg at the knee; 2—cannot flex the knee, but can move the feet; 3—complete motor paralysis of the legs.

## Data Availability

The data presented in this study are available on request from the corresponding author due to privacy or ethical restrictions.
